# Characteristic MRI Features of Meckel’s Cave Venous Malformation: A Case Report

**DOI:** 10.7759/cureus.116760

**Published:** 2026-09-22

**Authors:** Ece Bicakci, Erhan Biyikli, Hediye Pinar Gunbey

**Affiliations:** 1 Radiology, Marmara Üniversitesi Eğitim ve Araştırma Hastanesi, Istanbul, TUR

**Keywords:** benign vascular lesions, case report, contrast-enhanced mri, meckel’s cave, mri imaging, venous malformations

## Abstract

Venous malformations of Meckel’s cave are exceedingly rare lesions, with previously reported cases either predating modern MRI or requiring histopathological confirmation. We present the case of a 70-year-old male who was referred to our institution following detection of a left Meckel’s cave lesion on MRI performed for headache and dizziness. MRI demonstrated a well-defined 21 × 12 mm lesion exhibiting marked T2 hyperintensity, T1 isointensity, absence of diffusion restriction on diffusion-weighted imaging, and progressive centripetal nodular enhancement following intravenous contrast administration. Balanced fast-field echo sequence confirmed the lesion’s location within Meckel’s cave. These imaging characteristics supported an imaging-favored, non-invasive diagnosis of Meckel’s cave venous malformation, and conservative management with follow-up was recommended. Recognition of this characteristic MRI pattern (T2 hyperintensity, facilitated diffusion, and progressive centripetal enhancement) supports differentiation of venous malformation from more common Meckel’s cave lesions such as schwannoma, meningioma, and epidermoid cyst, and may reduce reliance on unnecessary surgical intervention.

## Introduction

Meckel’s cave is a cerebrospinal fluid (CSF)-filled dural recess located in the medial middle cranial fossa, communicating posteriorly with the posterior cranial fossa via the porus trigeminus, and houses the trigeminal ganglion and proximal nerve roots. Various lesions may arise within this space, including schwannomas, meningiomas, epidermoid cysts, and perineural tumor spread, representing fewer than 0.5% of all intracranial tumors [1,2]. Venous malformations of Meckel’s cave are exceedingly rare, with only a limited number of cases reported in the literature, all of which were either diagnosed in the pre-modern multiparametric MRI era or required surgical and histopathological confirmation [3,4]. We did not identify a prior report describing non-invasive diagnosis based on contemporary multiparametric MRI characteristics alone and present this case to highlight the key imaging features that support an imaging-favored radiological diagnosis and may avoid unnecessary surgical intervention.

## Case presentation

A 70-year-old male patient with no known systemic disease or medication use presented with headache and dizziness. Neurological examination, including facial sensation, corneal reflex, and masticatory strength, was unremarkable, with no trigeminal nerve-related symptoms or deficits. He had initially undergone MRI at an external center, where a lesion was detected within Meckel’s cave, and he was referred to our institution for further evaluation.

MRI demonstrated a well-defined lesion measuring 21 × 12 mm within the left Meckel’s cave. The lesion exhibited marked T2 and fluid-attenuated inversion recovery hyperintensity (Figures 1a, 1b), and T1 isointensity relative to the adjacent brain parenchyma. Balanced steady-state free precession (bSSFP; B-FFE) sequence clearly delineated the lesion within Meckel’s cave (Figure 1c). Diffusion-weighted imaging (DWI) revealed no restricted diffusion, with corresponding high signal on the apparent diffusion coefficient map consistent with facilitated diffusion (Figures 1d, 1e).

**Figure 1:**
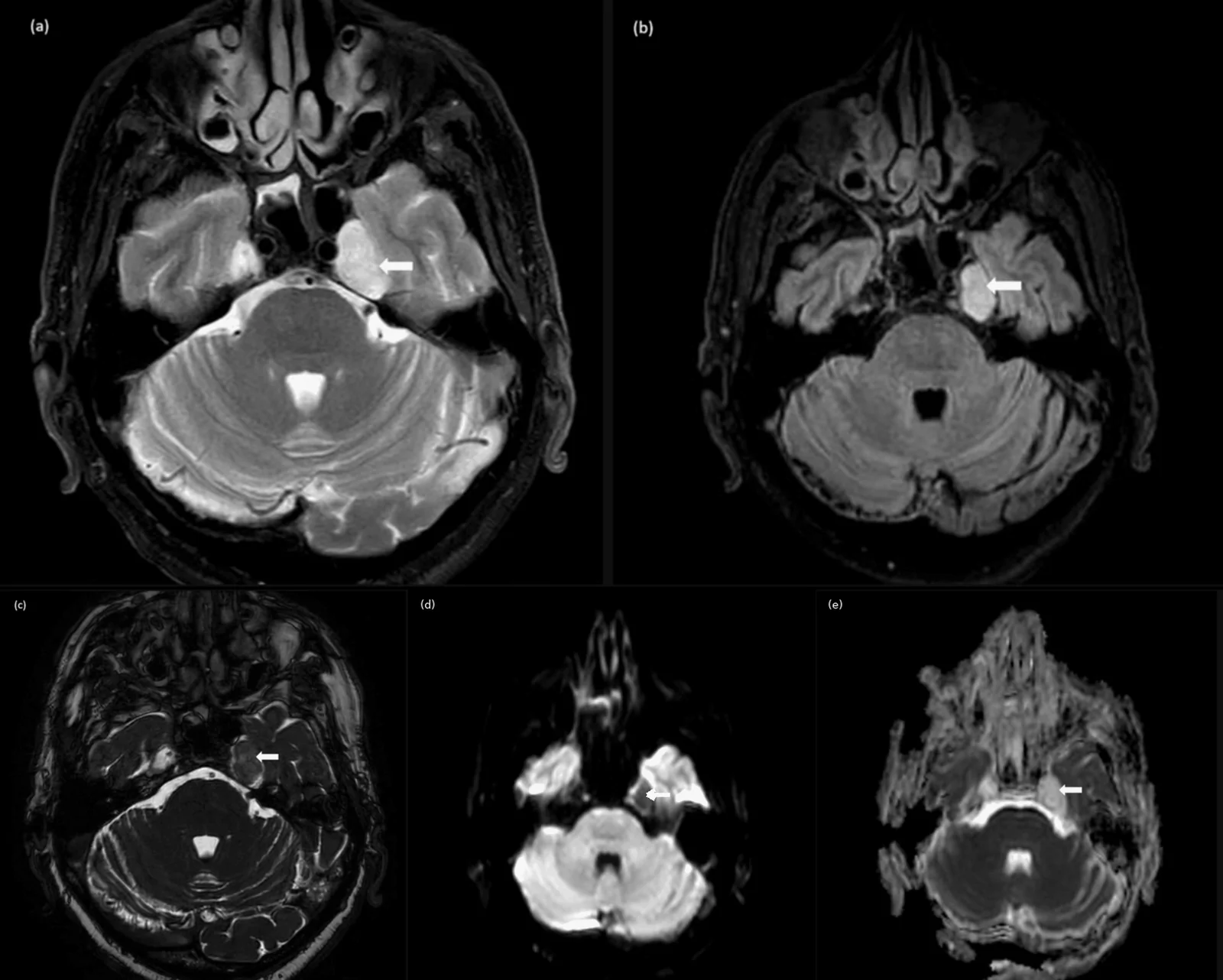
Multiparametric MRI of Meckel’s cave venous malformation. (a) Axial T2-weighted image demonstrating marked hyperintensity of the left Meckel’s cave lesion (arrow). (b) Fluid-attenuated inversion recovery sequence demonstrating correspondingly marked hyperintensity (arrow). (c) Balanced fast-field echo sequence delineating the lesion within the cerebrospinal fluid-filled cave in relation to trigeminal nerve roots (arrow). (d) Axial diffusion-weighted imaging (b = 1,000) demonstrating no visually apparent diffusion restriction (arrow). (e) Apparent diffusion coefficient map showing high signal consistent with facilitated diffusion, excluding restricted diffusion (arrow).

Following contrast administration, the lesion demonstrated a progressive, centripetal fill-in pattern (Figures 2a-2c).

**Figure 2:**
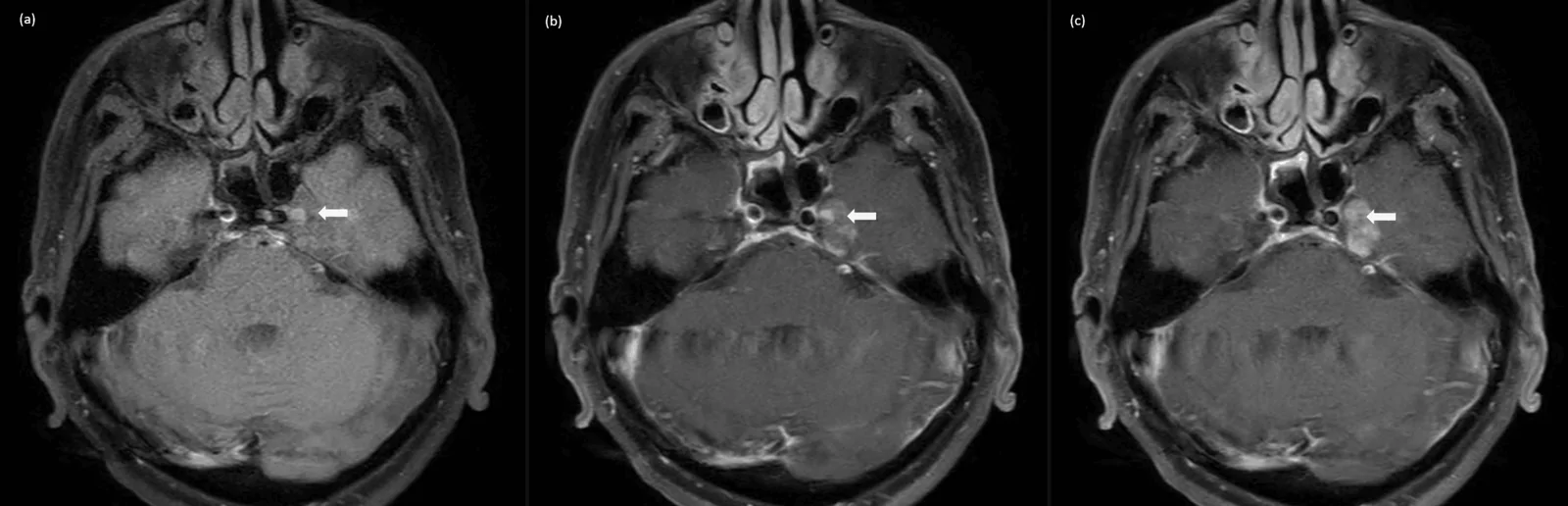
Progressive centripetal enhancement pattern of Meckel’s cave venous malformation. (a) Axial T1-weighted pre-contrast image demonstrating isointensity of the lesion. (b) Early post-contrast T1-weighted image showing peripheral nodular enhancement. (c) Delayed post-contrast T1-weighted image demonstrating progressive centripetal fill-in, characteristic of venous malformation.

Based on these imaging findings, an imaging-favored diagnosis of Meckel’s cave venous malformation was established. Given this diagnosis, conservative management with clinical and imaging follow-up was recommended. At the last clinical follow-up, the patient remained neurologically stable with no new or progressive symptoms; repeat imaging has not yet been performed but is planned as part of ongoing surveillance.

## Discussion

Meckel’s cave lesions represent a diagnostically challenging group. The most common entities include trigeminal schwannoma, meningioma, epidermoid cyst, and metastasis, all of which may present with overlapping clinical and imaging features [1,5]. Venous malformation is an exceedingly rare addition to this differential. The first reported case was described by Fehlings and Tucker in 1988, involving the gasserian ganglion and presenting with facial pain and trigeminal nerve deficit (then termed “cavernous hemangioma,” now recognized as venous malformation under current International Society for the Study of Vascular Anomalies (ISSVA) nomenclature) [3,6]. This case predated modern multiparametric MRI and required histopathological confirmation following surgical resection, as did the subsequent cases reported in the literature [4].

The MRI characteristics of our case are consistent with those of venous malformations at other intracranial extra-axial locations, particularly the cavernous sinus, with which Meckel’s cave shares dural venous continuity. Marked T2 hyperintensity, T1 isointensity, absence of diffusion restriction, and progressive centripetal nodular enhancement following contrast administration constitute characteristic imaging findings of this entity [7-9]. These features reflect the underlying vascular architecture of large, slow-flowing sinusoidal spaces, resulting in characteristic delayed centripetal fill-in. Jinhu et al. demonstrated that progressive contrast fill-in on conventional contrast-enhanced MRI was present in 95.2% of surgically confirmed cavernous sinus venous malformations (then termed “cavernous hemangiomas”), supporting its value as a key diagnostic feature [8]. Beyond the intracranial compartment, venous malformations are similarly well described across a wide range of extracranial head and neck sites, including the orbit, oral cavity, superficial and deep facial spaces, and supraglottic region, consistent with contemporary ISSVA-based classification of these lesions [6,10,11].

Trigeminal schwannoma, the most common Meckel’s cave tumor, typically demonstrates T1 hypointensity, T2 hyperintensity, and homogeneous or heterogeneous enhancement but lacks the progressive centripetal pattern [5]. Meningioma generally appears isointense on both T1 and T2 sequences with homogeneous enhancement and may demonstrate a dural tail sign [5]. Epidermoid cyst, while sharing T2 hyperintensity, is reliably distinguished by restricted diffusion on DWI, a feature absent in our case [12]. None of these entities replicate the combination of striking T2 signal approaching CSF intensity and progressive centripetal enhancement seen here [9], although progressive centripetal enhancement alone is not specific to venous malformation and may rarely be observed in meningioma or schwannoma; it is therefore best regarded as a supportive imaging clue rather than a definitive diagnostic criterion. The B-FFE sequence provided precise anatomical delineation of the lesion and its relationship to the trigeminal nerve roots. Importantly, venous malformations carry significant intraoperative hemorrhage risk [4,9], making preoperative radiological diagnosis valuable in avoiding unnecessary surgical exploration.

This case has inherent limitations. The diagnosis is based solely on imaging characteristics without histopathological confirmation, as the patient was managed conservatively. Additionally, while progressive centripetal enhancement is a characteristic feature of venous malformation, it is not entirely specific and may occasionally be seen in other lesions such as meningioma or schwannoma. The combination of marked T2 hyperintensity approaching CSF signal, absence of diffusion restriction, and progressive centripetal fill-in, considered together, supports an imaging-favored rather than definitive diagnosis.

## Conclusions

Meckel’s cave venous malformation is an exceedingly rare entity that poses a significant diagnostic challenge given its overlap with more common lesions such as trigeminal schwannoma, meningioma, and epidermoid cyst. The combination of marked T2 hyperintensity approaching CSF signal intensity, absence of diffusion restriction on DWI, and progressive centripetal nodular enhancement on contrast-enhanced MRI constitutes a characteristic imaging pattern that supports an imaging-favored, non-invasive diagnosis. Recognition of this pattern may spare patients the significant hemorrhagic risks associated with unnecessary surgical exploration of these highly vascular lesions. The favorable clinical course observed at follow-up supports conservative management with continued surveillance as a safe approach in imaging-favored cases such as this one.
